# Thiamine Diphosphate in Whole Blood, Thiamine and Thiamine Monophosphate in Breast-Milk in a Refugee Population

**DOI:** 10.1371/journal.pone.0036280

**Published:** 2012-06-29

**Authors:** Wolfgang Stuetz, Verena Ilona Carrara, Rose McGready, Sue Jean Lee, Hans Konrad Biesalski, François Henry Nosten

**Affiliations:** 1 Institute of Nutrition, Friedrich-Schiller-University, Jena, Germany; 2 Institute of Biological Chemistry and Nutrition, University of Hohenheim, Stuttgart, Germany; 3 Shoklo Malaria Research Unit, Mae Sot, Thailand; 4 Faculty of Tropical Medicine, Mahidol University, Bangkok, Thailand; 5 Centre for Clinical Vaccinology and Tropical Medicine, Churchill Hospital, Oxford, United Kingdom; Indiana University, United States of America

## Abstract

**Background:**

The provision of high doses of thiamine may prevent thiamine deficiency in the post-partum period of displaced persons.

**Methodology/Principal Findings:**

The study aimed to evaluate a supplementation regimen of thiamine mononitrate (100 mg daily) at the antenatal clinics in Maela refugee camp. Women were enrolled during antenatal care and followed after delivery. Samples were collected at 12 weeks post partum. Thiamine diphosphate (TDP) in whole blood and thiamine in breast-milk of 636 lactating women were measured. Thiamine in breast-milk consisted of thiamine monophosphate (TMP) in addition to thiamine, with a mean TMP to total thiamine ratio of 63%. Mean whole blood TDP (130 nmol/L) and total thiamine in breast-milk (755 nmol/L) were within the upper range reported for well-nourished women. The prevalence of women with low whole blood TDP (<65 nmol/L) was 5% and with deficient breast-milk total thiamine (<300 nmol/L) was 4%. Whole blood TDP predicted both breast-milk thiamine and TMP (R^2^ = 0.36 and 0.10, p<0.001). A ratio of TMP to total thiamine ≥63% was associated with a 7.5 and 4-fold higher risk of low whole blood TDP and deficient total breast-milk thiamine, respectively. Routine provision of daily 100 mg of thiamine mononitrate post-partum compared to the previous weekly 10 mg of thiamine hydrochloride resulted in significantly higher total thiamine in breast-milk.

**Conclusions/Significance:**

Thiamine supplementation for lactating women in Maela refugee camp is effective and should be continued. TMP and its ratio to total thiamine in breast-milk, reported for the first time in this study, provided useful information on thiamine status and should be included in future studies of breast-milk thiamine.

## Introduction

At the end of 1980s, thiamine deficiency was recognized as a major cause of infantile mortality in Maela refugee camp, north-western Thailand [1]. Following routine supplementary food rations (4 eggs and 500 g soybeans/wk) to all pregnant and post partum women, oral daily thiamine hydrochloride supplements (100 mg) were provided until delivery for those women with clinical signs of beriberi. In addition, thiamine hydrochloride (10 mg) was provided weekly to all lactating women. This supplementation program as well as intramuscular thiamine in suspected cases of deficiency, reduced the infantile mortality rate by 80%, but did not prevent biochemical thiamine deficiency (58% with erythrocyte transketolase activity <1.20) and low breast-milk thiamine (median of 379 nmol/L, 17% with thiamine <300 nmol/L) in women at 3 months postpartum [2]. Since 1998, additional weekly supplementary food rations (500 g split mung beans and 300 g dried fish) and daily thiamine mononitrate (100 mg) have been provided to all pregnant and post partum women attending the antenatal care consultations in the clinics of the Shoklo Malaria Research Unit (SMRU). The general monthly food ration for adults in Maela refugee camp included rice (15 kg), split mung beans (1.5 kg), fermented fish (1 kg), iodized salt (300 g), soybean oil (1 litre) and dried chillies (125 g).

Thiamine in human tissue and body fluids occurs mainly as thiamine diphosphate (TDP), thiamine monophosphate (TMP) and free thiamine [3], [4]. TDP, the most abundant thiamine derivative, is well described as a cofactor of several important enzymes (alpha-ketoglutarate dehydrogenase complex, pyruvate-dehydrogenase complex, transketolase), whereas TMP and thiamine are thought to be simple intermediates with as yet no specific role found. TMP was reported in cerebrospinal fluids with higher concentrations than those of TDP [5], [6].

The standard technique for assessing thiamine status is the measurement of erythrocyte transketolase activity (ETKA). This indirect approach lacks sensitivity by only detecting deficiency and cannot be applied for the analysis of thiamine in breast-milk. Direct measurement of TDP in whole blood or erythrocytes by HPLC with fluorometric detection of its thiochrome derivative was shown to be a more sensitive and specific index of thiamine nutrition than using ETKA [7], [8]. TDP, the principle biologically active form of thiamine, represents >80% of all thiamine in whole blood [9], [10]. The measurement of thiamine content in breast-milk gives valuable information on the existence of thiamine deficiency in a community [11]. We analysed TDP in whole blood and thiamine in breast-milk by HPLC to assess thiamine status of lactating mothers from Maela refugee camp.

## Material and Methods

### Objective, Study Population and Field Procedure

The study aimed to evaluate thiamine supplementation (100 mg daily) through HPLC and fluorometric measurement of TDP in whole blood and thiamine in breast-milk at 12 weeks post partum. Maternal characteristics and factors associated with low thiamine concentrations were also explored. This study was conducted at the antenatal clinics of the Shoklo Malaria Research Unit (SMRU) in Maela camp, 50 km north of Mae Sot on the Thai Myanmar border, and was part of a larger project to evaluate food rations and supplements for pregnant and post partum women. It was conducted according to the guidelines laid down in the Declaration of Helsinki and approved by the Ethics Committee of the Faculty of Tropical Medicine of Mahidol University (TM-IRB 04/2004) in Thailand and the Oxford Tropical Research Ethics Committee, University of Oxford (OXTREC 009-04), UK. Written informed consent was obtained from all women. Between June 2004 and August 2007, 636 breast-feeding mothers who delivered singleton normal babies were assessed for thiamine status at 12 weeks post partum.

Capillary blood for hematocrit measurements were done on site in Maela camp. Hematocrit values were used to derive hemoglobin levels [12]. Non-fasting EDTA-whole blood (5 ml) was collected between 10∶00 am and 12∶00 midday by venepuncture into vacutainers. Breast-milk samples (5–10 ml) were collected by manual expression into glass tubes (Pyrex). Whole blood and breast-milk were portioned into Eppendorf tubes and frozen at −20°C before transportation to the SMRU office in Mae Sot where they were stored at −80°C. Samples were then sent (on dry ice) to the University of Hohenheim, Stuttgart, Germany for the analysis on thiamine and its phosphate esters.

### Thiamine in Whole Blood and Breast-milk

Thiamine diphosphate (TDP) in whole blood (EDTA) and thiamine and thiamine monophosphate (TMP) in breast-milk samples were determined using precolumn derivatization, reversed-phase liquid chromatography and fluorescence detection as previously described [13] with modifications. In brief, 500 µl of EDTA whole blood or 500 µl of breast-milk was deproteinized by the addition of 7% (w/w) cold perchloric acid. 500 µl of clear supernatant was derivatized with 100 µl freshly prepared oxidation reagent (12 mM-K_3_Fe(CN)_6_ in 3.35 M-NaOH) and the ‘thiochrome reaction’ was stopped with 20 µl of 5 M phosphoric acid. Whole volume was transferred to autosampler vials and 20 µl was analysed on a Merck Hitachi HPLC (LaChrom) equipped with autosampler, column oven (set at 40°C), and fluorescence detector (excitation/emission set at 367/435 nm). The separation of thiamine and its phosphate esters was achieved using a 5 *µ*m analytical column (Grom-Sil 120 ODS-4 HE, 125×4 mm, Grom, Germany) and an isocratic mobile phase consisting of methanol (17.5% vol/vol for whole blood TDP and 27.5% vol/vol for breast-milk thiamine) and phosphate buffer (0.15 M, pH 7) at a flow rate of 0.8 ml/min. External calibration curves from TDP, TMP and thiamine hydrochloride standards (Sigma Chemicals) prepared by serial dilution into 0.1 M-HCl were used for quantification. For internal quality control, pools of whole blood and milk were analysed within each batch (n = 12) of samples giving inter-batch coefficients of variation for whole blood TDP of <7% and for TMP and thiamine in breast-milk of <4.5%. The limit of quantification of the method (signal-to-noise ratio of 10) for thiamine and its phosphate esters was <5 nmol/L. Whole blood TDP<65 nmol/L adapted to the lower range of 95% CI reference values reported for women (<30 years) from Japan [9] and breast-milk thiamine <300 nmol/L [11] were considered indicative of low whole blood TDP and thiamine deficiency, respectively.

### Breast-milk Samples from Maela 1995–1996

Results of thiamine concentrations of 17 breast-milk samples from a previous study in Maela refugee camp in 1995/6 [2], which were analysed by HPLC after enzymatic digestion of the thiamine derivatives to free thiamine and its oxidation to fluorescent thiochrome, were used to compare with the breast-milk total thiamine of the present study.

### Breast-milk Samples from Stuttgart

To confirm our finding of thiamine monophosphate (TMP) in breast-milk, 6 lactating mothers (in week 10–15 post partum) from Stuttgart (contacted via a scientist working at our institute), Germany, kindly consented to provide milk samples by manual expression, following verbal explanation. The age of the women ranged between 30 and 41 years and none of mothers reported to take thiamine supplements. Breast-milk was collected using the same protocol as in Maela camp. Samples were immediately stored on ice and transported to the University of Hohenheim in order to freeze (at −80°C) until HPLC analysis.

The presence of TMP itself was confirmed in milk pools (from ‘Stuttgart’ and Maela samples) through its hydrolysation to ‘free’ thiamine using clara- and taka-diastase. Deproteinized milk samples (400 µl) were incubated overnight (at 38°C) with 4.5 M sodium actetate (100 µl → pH 4.5) and a mixture of clara- and taka-diastase (100 µl, each 50 mg per ml); following centrifugation, supernatants were oxidized with potassium ferricyanide and analysed by HPLC and fluorometric detection as described above.

The presence of TMP and thiamine in the breast-milk pools were finally confirmed by liquid chromatography mass spectrometry (LC-MS) via electrospray ionization (ESI). Deproteinized milk pools (1∶1 with ethanol) and standards (TMP, thiamine) were scanned (range: *m/z* 250–450) and analysed on recorded single ion masses (*m/z* 343 and 381 for TMP; *m/z* 265 for thiamine) using an API 2000 mass spectrometry detector (MDS SCIEX) operated in positive ionization mode; a Grom-Sil analytical column (see above) and an isocratic mobile phase (0.3 ml/min) of methanol-water (10∶90 vol/vol) were used for separation.

### Statistical Analysis

Maternal characteristics were described by their medians (inter-quartile range). For categorical variables frequencies were reported. Normally distributed variables were described using means (SD). Data that were not normally distributed (thiamine, total thiamine in breast-milk, TMP ratio) were transformed using square root and square transformations; they were presented by their geometric mean (95% CI). Correlation between two variables was assessed using Pearson’s product-moment correlation coefficient (r^2^). Multivariate linear and logistic regression models with a forward stepwise approach were applied to identify independent predictors and respective deficiencies of whole blood TDP and breast-milk thiamine. The following covariates (at 12 weeks post partum) were examined: maternal age, body mass index (BMI), parity, smoking status (yes/no), regular chewing of betel nut (areca) (y/n), daily consumption of fermented fish (y/n), years of the living in the Maela camp, women’s religion (Christian, Buddhist or Muslim), days of provided supplements at the time of blood draw, hemoglobin levels, anemia defined as hemoglobin levels <120 g/L (y/n), whole blood TDP (in nmol/L or <65 nmol/L) and the TMP to total thiamine ratio (in % or ≥the median of 63%). All variables that were significant (*P*<0.05) from the univariate analysis were included and only significant predictors (*P* values <0.05) were retained in the final models. The fit of linear regression models were checked by visual examination of the distribution of the residuals; appropriate fit of logistic regression models was confirmed using the Hosmer-Lemeshow goodness-of-fit test.

The Student’s *t* test was used to compare mean breast-milk total thiamine with the concentrations measured in a previously study (1995/6) in Maela camp. Whole blood TDP and thiamine concentrations in milk were compared between women with a TMP to total thiamine ratio in breast-milk of less than the median of 63% with those who had ≥63%. All statistical analysis was carried out using SPSS software (SPSS Inc., Chicago, IL; Version 11.5).

## Results

The 636 women were of normal BMI with a median length of stay in the refugee camp of 8.8 years (Table 1). Thiamine in breast-milk was composed of thiamine monophosphate (TMP) in addition to free thiamine with mean proportions of 63% and 37% of total thiamine, respectively (Table 1). TDP was detected only in negligible small amounts (in average <1% of total thiamine, data not shown). The finding of breast-milk containing the mono-phosphorylated form of thiamine was confirmed in breast-milk samples (n = 6) of non-supplemented women from Stuttgart, Germany: mean total thiamine of 757±153 nmol/L with a mean proportion of TMP per total thiamine of 57±23%. The existence of TMP in breast-milk was proven through its enzymatic hydrolysis in pooled samples from Maela and Stuttgart: ‘free’ thiamine increased by the corresponding TMP concentration in the respective samples. The analysis by mass spectrometry in the selected ion monitoring mode confirmed TMP (*m/z* 343; *m/z* 381) and thiamine (*m/z* 265) as well as higher TMP than thiamine concentrations in the milk pools from Maela and Stuttgart.

**Table 1:** Characteristics of the study population, whole blood TDP and breast-milk thiamine at 12 weeks post partum ^1–6^.

Characteristics	*N* = 636
Age (y)^1^	26.8 (21.0, 31.8)
Parity (*n*)	3 (2, 4)
BMI (kg/m^2^)	21.7 (20.0, 23.5)
In Maela (y)	8.8 (5.8, 10.9)
Thiamine supplements (d)^2^	286 (258, 303)
Smokers (%)	27
Daily betel nut (%)	25
Fish paste, 2–3x/d (%)	57
Buddhist (%)	43
Christian (%)	40
Muslim (%)	17
**Whole blood**	***N*** ** = 636**
TDP (nmol/L)^3^	129.8±42.1
<65 nmol/L (%)	4.9
Hemoglobin (g/L)	125.7±11.8
<120 g/L (%)	31
TDP/Hb (ng/g)^4^	477.2±154.2
**Breast-milk**	***N*** ** = 636**
TMP (nmol/L)	445.7±147.7
Thiamine (nmol/L)^5^	297.0 (278.9–315.8)
Total thiamine (nmol/L)^6^	755.4 (730.4–780.7)
<300 nmol/L (%)	4.1
TMP per total thiamine (%)	63.5 (62.2–64.7)

1 Median (interquartile range), all such values.

2 Days of provided thiamine mononitrate (daily 100 mg).

3 Mean and standard deviation, all such values.

4 TDP/Hb, thiamine diphosphate per hemoglobin.

5 Geometric mean (95% confidence interval), all such values.

6 Total thiamine, sum of thiamine and thiamine monophosphate (TMP).

**Figure 1:**
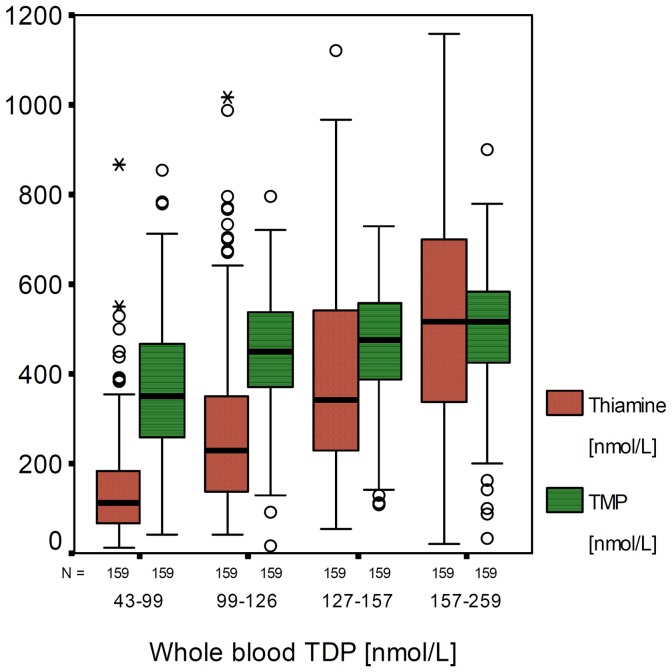
Thiamine and thiamine monophosphate (TMP) in breast-milk by quartiles of whole blood thiamine diphosphate (TDP).

Five percent of all studied women had low whole blood TDP (<65 nmol/L) and four percent had deficient breast-milk thiamine (total thiamine <300 nmol/L) (Table 1). Whole blood TDP was positively correlated with hemoglobin and was significantly higher in Christians than in Buddhists or Muslims (Table 2). Neither the chewing of betel nut, daily consumption of fermented fish nor the days of provided thiamine supplements post-partum at time of blood draw were associated with concentrations of whole blood TDP. Thiamine in breast-milk was positively correlated with TMP (r = 0.252, p<0.001), and both thiamine and TMP were strongly associated with the concentration of whole blood TDP (R^2^ = 0.36 and 0.10, respectively): TMP increased 1.12 nmol/L and [square root] thiamine increased 0.10 nmol/L per nmol/L increase in whole blood TDP (Table 2). BMI was negatively associated with thiamine but significantly positively correlated with TMP in breast-milk. Betel nut chewing was inversely associated with breast-milk TMP (Table 2). Independent predictors of total breast-milk thiamine included whole blood TDP and the ratio of TMP to total thiamine (R^2^ = 0.37 and 0.12, respectively); [square root] total thiamine in breast increased (+0.06, 95% CI 0.05 to 0.07) per unit increase in whole blood TDP and decreased (−0.13, 95% CI −0.15 to −0.11) per unit increase in TMP ratio. A higher ratio of TMP to total breast-milk thiamine than 63% (median of the present study) indicated lower thiamine status (Table 3). Women with an equal or higher TMP proportion of 63% had a significantly lower whole blood TDP and lower free thiamine and total thiamine in breast-milk than those with a TMP proportion of <63%; the concentrations of breast-milk TMP were similar in both groups. Logistic regression analysis revealed low whole blood TDP, TMP to total thiamine ratio ≥63% and smoking as independent risk factors of deficient breast-milk thiamine (Table 4); high TMP ratio (≥63%) was associated with a 4- and 7.5-fold higher risk of deficient breast-milk thiamine and low whole blood TDP, respectively. There was a limited increase of TMP but a significant linear increase of free thiamine in breast-milk with increasing concentrations of whole blood TDP (Figure 1), suggesting whole blood TDP had more impact on free thiamine than on TMP in milk.

**Table 2:** Multivariate linear regression analysis on TDP in whole blood and thiamine, TMP, and total thiamine in breast-milk, *N* = 636^ 1–6^.

	β coefficient^2^	95% CI	*P* value	*R* 2 change
**WB TDP** 3 (nmol/L)				
Hemoglobin (g/L)	0.62	0.33, 0.91	<0.001	0.025
Christian ( = 1)	14.2	7.70, 20.7	<0.001	0.023
In Maela (years)	−1.24	−2.09, −0.40	0.004	0.011
Age (years)	0.65	0.18, 1.11	0.006	0.011
**[SR]** 4 **Thiamine** (nmol/L)				
WB TDP (nmol/L)	0.10	0.09, 0.11	<0.001	0.364
BMI (kg/m^2^)	−0.24	−0.39, −0.08	0.002	0.007
Age (years)	0.08	0.02, 0.14	0.011	0.006
**TMP** (nmol/L)^5^				
WB TDP (nmol/L)	1.12	0.87, 1.38	<0.001	0.100
BMI (kg/m^2^)	6.40	2.49, 10.3	0.001	0.013
Betel nut, daily ( = 1)	−34.7	−59.5, −9.84	0.006	0.010
**[SR] Total thiamine** 6 (nmol/L)				
WB TDP (nmol/L)	0.06	0.05, 0.07	<0.001	0.373
TMP ratio (%)	−0.13	−0.15, −0.11	<0.001	0.123

1 Independent predictors are listed in the order they were entered in the forward models.

2 Indicates the increase in outcome per unit increase in risk factor.

3 WB TDP, whole blood thiamine diphosphate.

4 SR, Square root.

5 TMP, thiamine monophosphate.

6 Total thiamine, sum of thiamine and TMP.

**Table 3:** Whole blood TDP and thiamine, TMP and total thiamine in breast-milk of women with a low (<63%) compared to those with a high (≥63%) TMP to total thiamine ratio ^1–3^.

	TMP<63% (n = 323)	TMP≥63% (n = 313)	*P* value
**WB TDP** 1 **(nmol/L)**	149.3±38.9	109.7±35.3	<0.001
***Breast-milk***			
**TMP** 2 **(nmol/L)**	443.7±153.8	447.9±141.4	0.721
**Thiamine (nmol/L)**	502.4 (477–528)	141.4 (132–151)	<0.001
**Total thiamine** 3 **(nmol/L)**	946.0 (911–981)	581.1 (557–605)	<0.001

1 WB TDP, whole blood thiamine diphosphate.

2 TMP, thiamine monophosphate.

3 Total thiamine, sum of thiamine and TMP.

**Table 4:** Risk factors for deficient breast-milk total thiamine (<300 nmol/L; n = 26/636) and low whole blood TDP (<65 nmol/L; n = 31/636)^ 1–3^.

	Odds ratio	95% CI	*P* value
**Total thiamine** 1 **<300 nmol/L**			
WB TDP^2^<65****nmol/L	14.8	5.76, 38.2	<0.001
TMP^3^ ratio ≥63%	4.02	1.30, 12.4	0.016
Smoker ( = 1)	2.60	1.09, 6.22	0.031
**Whole blood TDP<65 nmol/L**			
TMP≥63%	7.53	2.60, 21.8	<0.001

1 Total thiamine, sum of TMP and thiamine.

2 WB TDP, whole blood thiamine diphosphate.

3 TMP, thiamine monophosphate.

The mean (95% CI) breast-milk total thiamine in the present study was significantly higher than in samples from Maela camp collected in 1995–1996: 755 (730, 781) vs. 430 (342, 529) nmol/L, p<0.001.

## Discussion

This present study aimed to analyze a vitamin supplementation program by measuring thiamine in breast-milk. A high peak appeared in all samples during the chromatography. This peak was thiamine monophosphate (TMP). This was unexpected and as far as we are aware the first time, that thiamine in human milk has been reported to consist of TMP in addition to free thiamine. TMP contributed to a higher extent to total thiamine (63%) than did free thiamine. Previous studies failed to report breast-milk TMP because thiamine was analysed as total thiamine by a microbiological assay [14] or in hydrolysed samples following manual [15], [16], [17] or HPLC-coupled [2], [18] fluorometric detection of thiochrome. The pre-treatment with enzymes to hydrolyse thiamine phosphate esters before derivatization to fluorescent thiochrome reveal the information only on total thiamine as a sum of ‘free’ thiamine content. Breast-milk samples of the present study were not hydrolysed and thus allowed the detection of thiamine as well as of TMP as their thiochrome derivates after HPLC separation. We confirmed the presence of TMP with a mean ratio to total thiamine of 57% in breast-milk in an independent set of samples (n = 6) collected in Stuttgart, Germany. In animal tissues, TMP is described as a product of the enzymatic hydrolysis of TDP with as yet no known specific metabolic role [19]. TMP as the only thiamine phosphoric ester with an amount of about 60% of total thiamine has been reported in the cerebrospinal fluid of different mammals including human [5]. Higher concentrations of TMP than other thiamine derivatives (including TDP) have been described for human kidney, embryonic tissues and gynecological specimens [20]. In the present study, TMP and its ratio to total thiamine in breast-milk was related to thiamine status. A high TMP to total thiamine ratio was associated with low whole blood TDP and deficient total thiamine concentrations in breast-milk. The limited increase in breast-milk TMP over the wide range of increasing whole blood TDP could depend on an active transport and saturation mechanism for TMP as it had been previously suggested for TMP in cerebrospinal fluid of healthy individuals [21]. Results suppose an active transport of TMP for substantial thiamine concentration in breast-milk. Three transporter proteins of the SLC19 gene family are described for the homeostasis of thiamine substrates [3], [22]. These include the reduced folate carrier (RFC) which transports folate as well as phosphorylated thiamine (TDP, TMP), and the thiamine transporters 1 and 2 (THTR-1, THTR-2) which are competent for the transfer of free thiamine. The RFC could be responsible for basic thiamine by means of TMP secretion in breast-milk and might be an important transport route among lactating mothers with low thiamine status. Further genetic variants and factors such as BMI and betel nut chewing may affect the proportion of RFC-mediated TMP secretion. Chewing of betel nut negatively impacts TMP but not thiamine concentrations while BMI was positively associated with TMP in breast-milk. However, further research is needed to gain insight to the physiology of TMP secretion and to confirm our finding of TMP ratio as an additional indicator of thiamine status.

Whole blood TDP in post partum women from Maela was in the upper range of concentrations reported for adult females of Norway and Japan [9], [23] or healthy American or Dutch adults [10], [13]. In north-eastern Thailand betel nut chewing as well as the consumption of raw fermented fish resulted in a biochemical thiamine deficiency even in the presence of adequate thiamine intakes [24]. In lactating women from Maela camp, low hemoglobin but not betel nut chewing nor the consumption of fermented fish paste were associated with low whole blood TDP. The regular consumption of fish paste did not affect thiamine status suggesting that: thiaminases were already destroyed by cooking, the fish paste did not contain thiaminases, or had an untraceable impact on the supplementation program due to the high doses of thiamine provided (daily 100 mg thiamine mononitrate compared to recommended daily thiamine intake of 1.5 mg for lactating women [25]). Betel nut consumption in this population has been explored in detail in over 4,900 pregnancies with no adverse impact on maternal or infant outcome postulated to be due to the use of ripe areca nut in conjunction with piper betel leaf, low rates of excessive consumption and thiamine supplementation (Chue AT *et al.*, unpublished results). Demonstrated here, is that compliance to thiamine supplements seems to eclipse the possible implication by betel nut and fish paste as ‘anti-thiamine’ factors. The lack of correlation between concentrations of whole blood TDP and total days of thiamine mononitrate until 12 weeks maybe explained by a high compliance to provided supplements post partum and during pregnancy [26]. The high mean whole blood TDP in post partum seemed to be the consequence of a prior increase of whole blood TDP during pregnancy and the ongoing provision of thiamine supplements after delivery. Thiamine and thiamine monophosphate in breast-milk were positively correlated with whole blood TDP and depended on dietary intake and thiamine status of the mother.

There were limitations to this study as we did not measure erythrocyte transketolase activity (ETKA) for comparison with previous data from Maela camp [2]. The collection, preparation (washing steps) and storage (liquid nitrogen) of erythrocytes for the determination of ETKA is time-consuming and difficult to handle, particularly with the large sample size involved here. Nevertheless a high correlation between ETKA and TDP in erythrocytes or whole blood as well as the higher sensitivity to detect thiamine deficiency by direct measurement of TDP is well described [7], [8]. Additional limits include the data on days of thiamine supplements which are what were supplied to the woman not what was actually ingested. As there was no significant change in provided food rations or habits (betel chewing) since 1995–1996 the observed increase in breast-milk total thiamine is most likely to be due general provision of high doses of thiamine (100 mg/day) for both pregnant and lactating women attending the SMRU clinics. Mean total thiamine in milk samples from Maela camp (755 nmol/L) was higher than reported for well-nourished Spanish (590 nmol/L) [17] and vitamin supplemented (1.7 mg B1/day) American mothers (706, 676 nmol/L) [15], [16]. Similar high amounts of thiamine were measured in mature breast-milk of Saudi women (800 nmol/L) [27]. The significant improvement in breast-milk thiamine collected 10 years apart from Maela refugee camp women suggests that the implementation of thiamine as a general pregnancy supplement (not only for symptomatic women) and in lactation at 100 mg/day not 10 mg/week was highly effective in this setting. Importantly high whole blood TDP in the post partum period and its significant positive correlation with total thiamine in breast-milk suggest a high compliance to provided thiamine supplements and an effective ante- and post-natal supplementation programme. This prevents thiamine deficiency and infantile beri-beri in breast-fed infants, the main reason for the previously observed high infant mortality rate in Maela camp [1].
